# An ancestral TMEM16 homolog from *Dictyostelium discoideum* forms a scramblase

**DOI:** 10.1371/journal.pone.0191219

**Published:** 2018-02-14

**Authors:** Thomas Pelz, Daniela R. Drose, David Fleck, Bastian Henkel, Tobias Ackels, Marc Spehr, Eva M. Neuhaus

**Affiliations:** 1 Pharmacology and Toxicology, Jena University Hospital, Friedrich Schiller University Jena, Jena, Germany; 2 Cluster of Excellence NeuroCure, Charité-Universitätsmedizin Berlin, Berlin, Germany; 3 Department of Chemosensation, Institute for Biology II, RWTH-Aachen University, Aachen, Germany; Indiana University School of Medicine, UNITED STATES

## Abstract

TMEM16 proteins are a recently identified protein family comprising Ca^2+^-activated Cl^-^ channels that generate outwardly rectifying ionic currents in response to intracellular Ca^2+^ elevations. Some TMEM16 family members, such as TMEM16F/ANO6 are also essential for Ca^2+^-dependent phospholipid scrambling. TMEM16-like genes are present in the genomes of most eukaryotic species, the function(s) of TMEM16 family members from evolutionary ancient eukaryotes is not completely clear. Here, we provide insight into the evolution of these TMEM16 proteins by similarity searches for ancestral sequences. All eukaryotic genomes contain TMEM16 homologs, but only vertebrates have the full repertoire of ten distinct subtypes. TMEM16 homologs studied so far belong to the opisthokont branch of the phylogenetic tree, which includes the animal and fungal kingdoms. An organism outside this group is *Dictyostelium discoideum*, a representative of the amoebozoa group that diverged from the metazoa before fungi. We here functionally investigated the TMEM16 family member from *Dictyostelium discoideum*. When recombinantly expressed in HEK293 cells, *Dd*TMEM16 induces phospholipid scrambling. However, in several electrophysiological experiments we did not find evidence for a Ca^2+^-activated Cl^-^ channel function of *Dd*TMEM16.

## Introduction

Ca^2+^-activated Cl^-^ channels play important roles in various physiological processes, from chloride secretion in epithelial cells to coupling of calcium elevation to membrane depolarization in smooth muscle cells and neurons. ANO1 and ANO2, members of the anoctamin (from ANion selective and eight (OCT) transmembrane segments [1]) or TMEM16 family of transmembrane proteins, have recently been described to exhibit very similar characteristic biophysical and pharmacological properties to endogenous Ca^2+^-activated chloride currents [1–3]. Knockdown of mouse *Ano1/Tmem16a* causes loss of Ca^2+^-activated Cl^-^ channel activity in cells from airway epithelium, biliary ducts, salivary glands, intestine, and blood vessel smooth muscle [1, 4]. ANO2/TMEM16B is specifically located to the cilia of sensory neurons in the olfactory epithelium [5, 6], and recombinantly expressed ANO2/TMEM16B exhibits channel properties closely resembling those of the native olfactory Ca^2+^-activated Cl^-^ channel [6, 7]. Disruption of *Ano2/Tmem16b* and *Ano1/Tmem16a* in mice abolished Ca^2+^-activated Cl^-^ currents in the olfactory and vomeronasal epithelium, respectively [8, 9]. In vertebrate photoreceptors, ANO2/TMEM16B is localized to synaptic terminals, suggesting that it is involved in the well-described membrane potential regulation via Cl^-^ currents [10].

The mammalian TMEM16/anoctamin protein family is composed of 10 members, ANO1-10 or TMEM16A-K. Despite ample evidence that the first molecularly characterized family members ANO1 and ANO2 form Ca^2+^-activated anion channels, function(s) of the other family members remain far less understood. It is unclear whether all TMEM16 subtypes are activated by Ca^2+^ and if there are other or additional regulators. Some family members have not primarily been described as anion channels. TMEM16C/ANO3 does not form homomeric ion channels, but controls the excitability of nociceptive neurons by modulating the activity of Slack, a Na^+^-activated K^+^ channel [11]. TMEM16E/ANO5 (initially named GDD1) is responsible for gnathodiaphyseal dysplasia [12], and does not exhibit cell surface Ca^2+^-activated Cl^-^ channel activity [13].

TMEM16F/ANO6 is expressed in many tissues and has been found to have different functions. It is required for Ca^2+^-regulated phospholipid scrambling in platelets [14], leading to externalization of phospholipids such as phosphatidylserine (PS) that are normally confined to the inner leaflet of the plasma membrane. Extracellular exposure of platelet PS is a key trigger for the initiation of blood clotting [15], and an important signal for phagocytic clearance of apoptotic cells [16, 17]. TMEM16F has been shown to form a small-conductance Ca^2+^-activated nonselective cation channel [18]. Other experiments showed that Ca^2+^-dependent phospholipid scrambling by TMEM16F coincides with ionic currents that are explained by ionic leakage [19]. TMEM16F/ANO6 was also shown to have anionic conductivity [20–22], and to be an essential component of the outwardly rectifying Cl^-^ channel in lymphocytes and in dendritic cells [20, 23]. Recombinantly expressed TMEM16C, TMEM16D, TMEM16G, and TMEM16J have also been suggested to work as scramblases [24]. In general, the TMEM16 family seems to be composed of Ca^2+^-gated Cl^-^ channels and Ca^2+^-dependent phospholipid scramblases. TMEM16F/ANO6 could fulfill both functions, or could be an ion channel that regulates another so far unknown phospholipid scramblase.

Analysis of available sequences showed that TMEM16 family members are apparently present in all animal genomes [25–27]. One TMEM16 family member from *Drosophila melanogaster*, Subdued (CG16718), forms a Ca^2+^-activated Cl^-^ channel when expressed in HEK293T cells [28]. TMEM16 family members have also been identified in other eukaryotes. A yeast TMEM16 homolog, Ist2, is involved in extracellular salt tolerance [29], which indicates that it could have ion channel function. By contrast, Ist2 was described as a tether connecting the endoplasmic reticulum and plasma membrane [30–32]. Purification and functional reconstitution of an ancestral TMEM16 homolog from *Aspergillus fumigatus* (*Af*TMEM16) showed that it has a dual-function, as an ion channel and a scramblase, with both functions being Ca^2+^-dependent [33]. A TMEM16 family member from the fungus *Nectria haematococca* (*Nh*TMEM16) operates as a Ca^2+^-activated lipid scramblase, but does not form an ion channel of large conductance [34]. Despite the observed lack of a large conductance upon expression of *Nh*TMEM16, lipid scrambling was shown to be associated with non-selective ion currents, similar to other scramblases [35]. This raises the possibility that the two processes represent a general functional feature of TMEM16 scramblases.

Since the three ancestral TMEM16 proteins studied so far seem to behave differently, more information on the general features of TMEM16 family members is required to gain a conceptual understanding of TMEM16 protein evolution and function. Here, we analyzed several databases for TMEM16 homologs and generated a phylogenetic tree showing the evolutionary relation of TMEM16 proteins throughout the eukaryotic domain, in plants, fungi, animals, down to single-celled eukaryotes. We found families composed of single members in plants, fungi, yeast and amoebozoa. *D*. *discoideum* is a social amoeba that serves as a valuable eukaryotic model organism for the study of membrane trafficking and signaling processes [36, 37], and for the analysis of the complex interactions between pathogenic bacteria and host cells [38]. We cloned and recombinantly expressed the only TMEM16 homolog from *D*. *discoideum* (*Dd*TMEM16). Recombinant *Dd*TMEM16 did not mediate Ca^2+^-activated currents, but instead induced phospholipid scrambling.

## Materials and methods

### Sequences

The following protein sequences were used for cloning and expression in this study. TMEM16 homolog from *Dictyostelium discoideum* termed *Dd*TMEM16 (Gene ID DDB_G0267752, dictyBase ID DDB0306499). The protein sequence of TMEM16A used was described as ANO1 *abcd* [3]. Mouse TMEM16F/ANO6 (AAH60732) sequence was described [18, 22].

### Expression analysis

RNA from *D*. *discoideum* was isolated using the RNeasy Mini Kit (Qiagen, 74104) and transcribed to cDNA using the First Strand cDNA Synthesis Kit (Thermo Scientific, K1612), each according to the manufacturer’s instructions. Expression of *Dd*TMEM16 was tested by RT-PCR using two specific primer pairs, *D*. *discoideum* tubulin was used as a control.

Dd_TMEM16_fwd1, GTGCTGCATCACCAATTTCACC

Dd_TMEM16_rev1, GCACGTTGTCTAGTTTTTGAAGTG

Dd_TMEM16_fwd2, CCATTGGTACCTTTTCAGTGGTTG

Dd_TMEM16_rev2, TGGTGATGCAGCACTAAACA

Dd_Tub_fwd, TCACTGCCAAAGGTGCCTCG

Dd_Tub_rev, ACCGATGAAGGTGACGGCCA

### Cloning and gene synthesis

The full length *Dictyostelium discoideum* TMEM16 coding sequence was amplified from *Dictyostelium* cDNA using the KAPAHifi PCR kit (Peqlab, 07-KK2100-01) with standard buffers and protocols. The PCR product was used as template for a second PCR to introduce flanking restriction sites, and the new product was cloned into pcDNA3.1 vector (Life Technologies, V790-20) carrying a GFP reporter sequence to generate a C-terminal fusion construct. All cloned sequences were verified by sequencing.

Dd_ TMEM16_fwd, ACCATGGAAGAAAGTAGTTATG

Dd_ TMEM16_rev, TTTATTTTCATCTTCATAATCAACATC

Dd_TMEM16_Hind_fwd, GCAAGCTTACCATGGAAGAAAGTAGTTATGATAATTTT

Dd_ TMEM16_Eco_rev, GCGAATTCTTATTTATTTTCATCTTCATAATCAAC

For the humanized version of *Dd*TMEM16 (DDB_G0267752) codon usage was optimized by Gene Art from Life Technologies, synthesized by Life Technologies, and subsequently cloned into the pcDNA6.2/EmGFP vector. The non-tagged channel was amplified from this plasmid and cloned into pIRES-EGFP vector (Clontech), a mammalian expression vector that allows expression of two separate genes of interest from the same bicistronic mRNA transcript.

### Cell culture and microscopy

HEK293 cells were seeded on glass coverslips in tissue culture dishes 35×10 mm (Sarstedt, 83.1800) and grown to 90% confluence for 48 h in DMEM supplemented with GlutaMax (Life Technologies), 10% fetal calf serum and 1% penicillin/streptomycin (PAA). Confluent cells were transfected with Turbofect (Thermo Scientific) according to the manufacturer’s instructions. After 24 h of protein expression, cells were washed with PBS buffer and fixed with 4% PFA for 15 min at room temperature. Afterwards, cells were washed with PBS buffer and mounted on microscopy slides with ProLong Gold Antifade (Sigma, 36930). Localization of *Dd*TMEM16-GFP protein in HEK293 cells was analyzed by imaging fluorescence with a Leica SPE confocal microscope (Leica, TCS SPE).

### Western blot

HEK293 cells were seeded in cell culture flasks (Thermo Scientific, 134381), grown and transfected as described before. After 24 h cells were washed and homogenized in ice cold buffer (5 mM Tris-HCl, 300 mM sucrose, 0.1 mM EDTA, Complete protease inhibitor cocktail (Roche), pH 7.4). Cell debris was removed by centrifugation (1500g, 15 min) and the supernatant was centrifuged for 35 min at 20,000 g for membrane protein enrichment. The pellet was resuspended in solubilization buffer (20 mM Tris-HCl, 10% (v/v) glycerol, 100 mM ammonium sulfate, 1% (w/v) CHAPSO (Sigma, C3649), Complete protease inhibitor cocktail, pH 7.4) and for all samples same amounts of proteins were diluted in 10 μl of SDS sample buffer (0,02% (w/v) bromophenol blue, 20% (v/v) glycerol, 4% (w/v) SDS, 200 mM DTT, 125 mM Tris-HCl, pH 6.8), separated by SDS-PAGE and blotted onto nitrocellulose membrane (GE Healthcare, 10401196). Proteins were detected by a specific antibody against the GFP tag (Roche, 11814460001) and HRP-coupled secondary antibody (Biorad, 170–6516). The signal was visualized using ECL Select reagent (RPN2235, Amersham) and a digital luminescence detection system (Vilber Lourmat, Fusion FX7) or x-ray film (Super RX-N, Fujifilm).

### Labeling of cell surface proteins

Cell surface proteins from transfected HEK293 cells were isolated using the Pierce Cell Surface Protein Isolation Kit (Thermo Scientific, 89881) according to the manufacturer’s instructions. In brief, primary amines of cell surface proteins were labeled with Sulfo-NHS-SS-Biotin, then cells were lysed and labeled protein was purified with streptavidin agarose beads. Aliquots for total protein input controls were removed from clarified lysates after cell lysis. For the labeled protein Western blot, sample volumes were adjusted to the respective input controls.

For imaging analysis, transfected cells were grown on glass coverslips and cell surface proteins were biotinylated using the above kit. After washing with PBS, cells were fixed with 4% PFA for 15 min, washed again with PBS and blocked for 1 h with blocking solution (1% gelatine, 0.1% Triton X-100). Cells were then incubated with Alexa Fluor 568-conjugated streptavidin (Thermo Scientific, S11226) in blocking solution for 1 h and afterwards washed with PBS. Coverslips were mounted as described above and specimens were subjected to confocal microscopy. Images were examined for TMEM16/streptavidin-A568 localization using Fiji v1.49k (plot profile tool). Resulting gray values were normalized to the maximum value of each channel and graphs were plotted with Graphpad Prism v6.01.

### Sequence alignment

**Fig 1:**
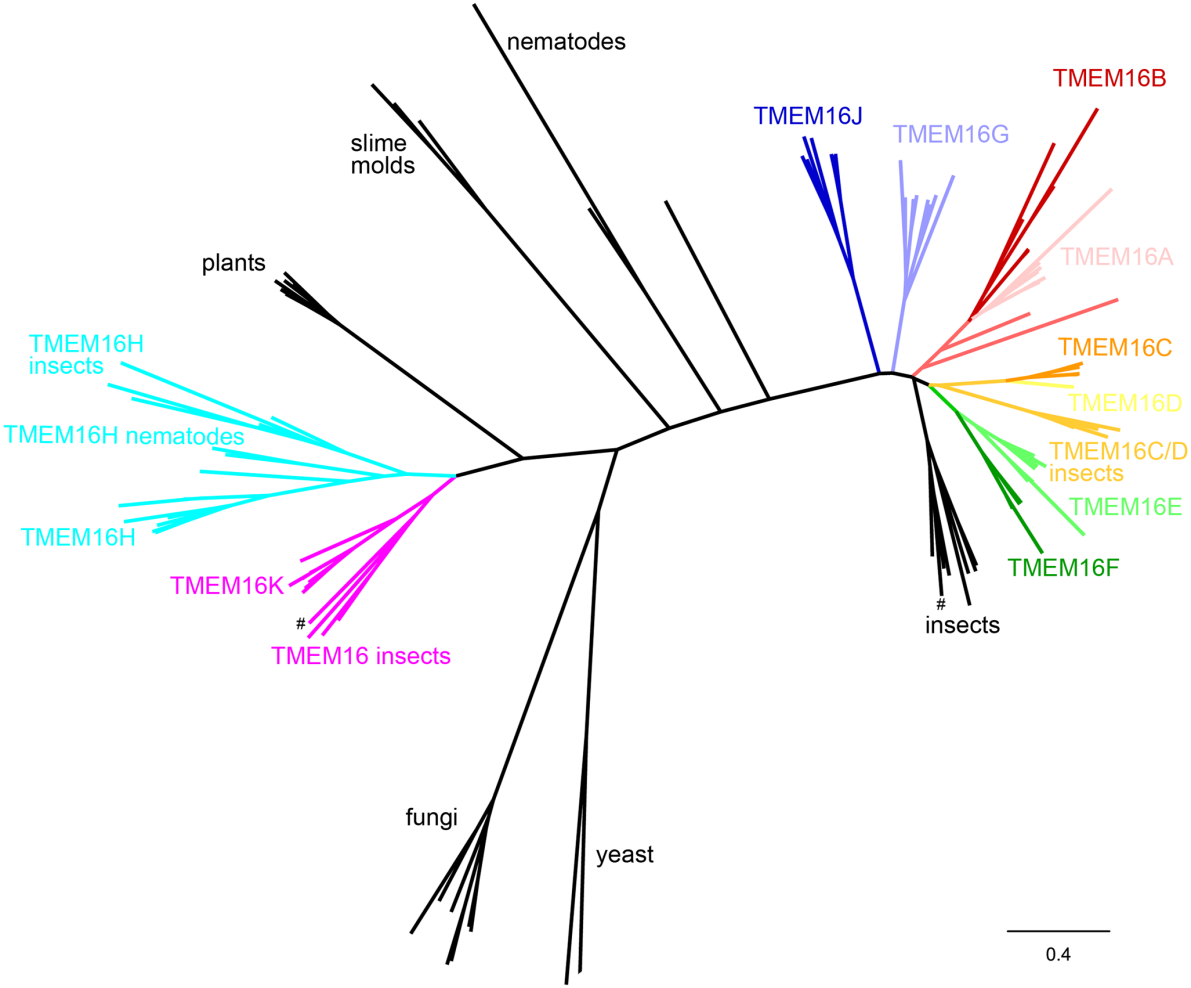
Phylogenetic analysis. Phylogenetic tree of TMEM16/ANO sequences from diverse eukaryotic species. TMEM16A-K/ANO1-10 from vertebrates are differently colored. TMEM16 homologs from insects are labeled and appear in the same color as the respective vertebrate branch where appropriate. In cases where no clear-cut classification to the vertebrate groups is possible, sequence clusters are colored black and labeled, # marks two functionally characterized TMEMs from *Drosophila melanogaster*, Axs (TMEM16K/ANO10) and Subdued (black labeled insect branch). Scale bar, expected fraction of amino acid substitution.

The data set in this study contained 450 sequences (Fig 1, S1 Sequences). TMEM16-like sequences were searched using tblastp and the mouse TMEM16A protein sequence in the NCBI database, species included in the alignment were from chordates (mammals, bony fish, reptiles, amphibia, cephalochordata), hemichordates, invertebrates (nematoda, arthropoda, cnidaria), land plants, algae, fungi, and amoebozoa. Sequences were aligned using CLC workbench (http://www.clcbio.com/). The phylogenetic tree was generated with CLC workbench and processed with FigTree (http://tree.bio.ed.ac.uk/software/figtree/).

### Chemicals/solutions for electrophysiological recordings

a) Extracellular solution (**S**_**1**_) containing (in mM): 145 NaCl, 5 KCl, 1 CaCl_2_, 1 MgCl_2_, 10 HEPES; pH = 7.3 (adjusted with NaOH); osmolarity = 300 mOsm (adjusted with glucose). b) TEA-containing extracellular solution (**S**_**2**_) containing (in mM): 124 NaCl, 15 TEACl, 1 MgCl_2_, 1 CaCl_2_, 10 HEPES; pH = 7.3; osmolarity = 300 mOsm. c) Reduced chloride solution (**S**_**3**_) containing (in mM): 119 Na gluconate, 15 TEACl, 1 Ca gluconate, 1 Mg gluconate, 5 CsCl, 10 HEPES; pH = 7.3 (NaOH); osmolarity = 300 mOsm. d) Pipette solution (**S**_**4**_) containing (in mM): 138 CsCl, 2 CsOH, 1 EGTA, 10 HEPES, 1 MgATP, 0.5 NaGTP, 0.3 CaCl_2_; pH = 7.1 (CsOH); osmolarity = 290 mOsm. e) Elevated Ca^2+^ pipette solution (**S**_**5**_) containing (in mM): 138 CsCl, 2 CsOH, 1 EGTA, 10 HEPES, 1 MgATP, 0.5 NaGTP, 1.51 CaCl_2_ (200 μM free Ca^2+^); pH = 7.1 (CsOH); osmolarity = 290 mOsm. If not stated otherwise, chemicals were purchased from Sigma (Schnelldorf, Germany). Final DMSO concentrations were ≤ 0.1%. Stimuli and pharmacological agents were applied via an 8-in-1 multi-barrel ‘perfusion pencil’ [39].

### Electrophysiology

All experiments were performed in whole-cell recording configuration. HEK293T cells were transferred to the stage of an inverse video-microscope (DMI 4000B, Leica Microsystems). Cells were continuously superfused with solution **S**_**1**_ (~3 ml/min; gravity flow) at room temperature. Patch pipettes with a resistance of ~5 MΩ were pulled from borosilicate glass capillaries with filament and fire-polished ends (1.50 mm OD / 0.86 mm ID; Science Products, Hofheim, Germany) on a PC-10 vertical two-step micropipette puller (Narishige Instruments, Tokyo, Japan) and fire-polished using a MF-830 microforge (Narishige Instruments). Depending on the experimental paradigm, recording pipettes were filled with **S**_**4**_ or **S**_**5**_ solution, respectively. An EPC-10 amplifier controlled by Patchmaster 2.67 software (HEKA Elektronik, Lambrecht/Pfalz, Germany) was used for data acquisition. Both pipette (C_fast_) and cell membrane capacitance (C_slow_) were monitored and automatically compensated throughout the experiment. Measured C_slow_ values served as an approximation of the cell surface area for normalization of current amplitudes (i.e. current density). HEK293T cells with unstable C_slow_ values and R_series_ ≥ 30 MΩ were not considered for further analysis. Theoretical liquid junction potentials were calculated using JPCalcW software and automatically corrected online. Signals were low-pass filtered (analog 3- and 4-pole Bessel filters in series) with a sampling rate of 10 kHz. Electric noise was suppressed by using a Hum Bug Noise Eliminator (Quest Scientific, Vancouver, Canada). Focal application of different stimuli and/or pharmacological agents was achieved by a software-controlled pressure-driven valve bank (TIB-14 S, HEKA Elektronik) connected to a ‘perfusion pencil’. Between recordings, cells were kept at a holding potential (V_hold_) of -60 mV. Experiments were performed using ramp protocols between -100 mV and +100 mV and a duration of 500 milliseconds with up to 150 repetitions. Continuous recordings were performed at V_hold_ of -80 mV.

### Ca^2+^ imaging

Ca^2+^ imaging in fura-2/AM loaded HEK293T cells was performed as previously described [22]. Briefly, dye-loaded cells were washed and imaged using an inverted microscope (Leica DMI4000B, Leica Microsystems) equipped for ratiometric live-cell imaging with a Visichrome polychromator system (Visitron Systems) for multiwavelength excitation, a 12-bit 1392 x 1040 CCD camera (CoolSnap EZ, Photometrics), and LAS MMAF software (Leica Microsystems). Cells were illuminated sequentially at 340 and 380 nm (1 Hz cycles) and average intensities within user-selected regions of interest were used to calculate f_340_/f_380_ intensity ratios.

Ca^2+^ concentrations [Ca^2+^] were calculated according to [40] using the equation [Ca^2+^] = *K*_d_ * (F_0_/F_s_) * [(R − R_min_)/(R_max_ − R)]. *K*_d_ is the fura-2 dissociation constant (224 nM), F_0_ and F_s_ are coefficients for free and Ca^2+^-bound fura-2, respectively (measured at 380 nm), and R_min_ and R_max_ were determined by applying 5 μM of ionomycin in [Ca^2+^]_zero_ (10 mM of EGTA) and saturating Ca^2+^ (10 mM). Values for R_min_, R_max_, and F_0_/F_s_ were 0.56, 0.85, and 1.88, respectively.

### Data analysis

All data were obtained from experiments performed on at least two days. Individual numbers of cells/experiments (n) are denoted in figure legends. For ramp recording analysis upon ATP stiumulation, 5 ramps before stimulation were averaged and subtracted from the peak amplitude ramp after stimulation. For recordings aimed to test for potential effects of high cytosolic Ca^2+^ concentrations (200 μM; **S**_**5**_-containing pipette solution), voltage ramp commands were repetitively recorded immediately after break-in and monitored over time. Currents were normalized to their maximum value and average current-voltage curves were plotted to compare results from transfected and non-transfected cells. If not stated otherwise, results are presented as means ± SEM and statistical analyses were performed using paired or unpaired t-tests (as dictated by data distribution and experimental design). Electrophysiological data were analyzed offline using Patchmaster 2.67 (HEKA Elektronik), IGOR Pro 6.31 (WaveMetrics, Lake Oswego, OR) and Excel (Microsoft, Seattle, WA) software.

### Phospholipid scrambling

HEK293 cell were grown on glass cover slips in 35×10 mm dishes and transfected after 24 hours as described before. 4×10^5^ and 2,5×10^5^ cells were seeded for 24 hours and 48 hours incubation after transfection, respectively. Cells were then washed twice with Annexin binding buffer ABB (10 mM HEPES, 140 mM NaCl, 2.5 mM CaCl_2_, pH 7.4) and stained with Alexa Fluor 568-conjugated Annexin V (1:100, Molecular Probes, A13202) and Hoechst 33342 (1:500 of a 10 mg/ml stock solution) for 15 min. Cells were washed once with ABB and immediately subjected to fluorescence microscopy (DMI6000, Leica Microsystems). For the analysis of Ca^2+^-dependent scramblase activity, cells 24 h post transfection were stimulated with either 3 μM A23187/Calcimycin (Sigma, C7522, 20 mM stock solution in DMSO) or vehicle only in ABB for 10 min. After three washes with ABB cells were stained as described above with ABB containing 5 mM CaCl_2_. From each sample three images were taken at different random positions with a 10X objective. Using Fiji v1.49k with the Cell Counter and Point Picker plugins the total number of cells (Hoechst 33342^+^) and the numbers of Annexin V^+^/GFP^+^ and Annexin V^+^/GFP^-^ cells were counted (non-blinded assessment). The numbers from the three images of one sample were added, amounting to a total of 4000–9000 cells per sample. Statistical analyses were performed using Graphpad Prism v6.01. For each condition at least three independent experiments were performed, as described in the figure legends. For the analysis of time-dependent phospholipid scrambling statistical significance was calculated by two-way analysis of variance (ANOVA) and Tukey’s multiple comparisons test. Indicated P values represent multiplicity-adjusted P values. To evaluate the A23187-induced phospholipid scrambling the background staining percentage value (vehicle control) was subtracted from the A23187 stimulation value for each independent experiment. Statistical significance of data was then analyzed by an unpaired two-tailed t test. For analysis of correlation between TMEM16 expression and Annexin V binding, regions of interest were drawn around the outlines of individual, stimulated, non-apoptotic Annexin V+/GFP+ cells from images described above, using Fiji v1.49k. For both channels mean gray values were quantified and background signals subtracted. Pearson coefficient and linear regression were calculated using Graphpad Prism v6.01.

## Results

### Phylogenetic analysis of the TMEM16 family

To understand the molecular evolution of TMEM16 family members, we started by performing a phylogenetic analysis across different species. Initial BLAST similarity searches (NCBI database) showed that the ten known TMEM16 sequences are found in all mammalian genomes with sufficient sequence coverage, while additional sequences do not seem to exist in mammals. Ten TMEM16 family members were also found in non-mammalian vertebrates. All bony fish genomes analyzed in this study showed several gene duplications which likely result from the 3R teleost-specific genome duplication events [41]. The complete phylogenetic tree of the data set is shown in Fig 1.

Vertebrate TMEM16 sequences group in two well-separated clusters, one being composed of TMEM16H and TMEM16K, the other one containing the remaining TMEM16 proteins. In the latter cluster, TMEM16A and TMEM16B build closely related subfamilies, as do TMEM16C/TMEM16D and TMEM16E/TMEM16F. TMEM16G and TMEM16J form separate branches, which are nonetheless more closely related to TMEM16A-F than to the TMEM16H/TMEM16K group. The genome of the lancelet amphioxus, a cephalochordate species, contains six TMEM16 genes. The number of genes in the animal lineage further declines in invertebrates. Insects have TMEM16H- and TMEM16K-like genes (with Axs from *Drosophila melanogaster* grouping with TMEM16K), and two to three genes (depending on the species) grouping with the TMEM16A-F cluster (one of these being *Drosophila* Subdued). One of two nematode TMEM16 proteins is a TMEM16H homolog (Anoh-2), the other gene (Anoh-1) branches off in between both clusters of vertebrate TMEM16 proteins.

We also identified TMEM16 homologs in essentially all other eukaryotic species with sufficient sequence information. Green plants, red and brown algae contain single TMEM16 genes. Fungi, including budding yeasts, contain 1 or 2 TMEM16 genes which do not group with the vertebrate families. The fungi group contains TMEM16 homologs from *Nectria haematococca* and *Aspergillus fumigatus*, for which structural and functional data are available. *Dictyostelium discoideum* and other slime molds are single-celled eukaryotes belonging to the amoebozoa, and contain single TMEM16 genes.

### Structural comparison of ancient TMEM16 homologs to vertebrate family members

A logo-plot of all TMEM16 sequences used in this study (S1 Fig) showed that the putative extracellular loops exhibit a lower degree of conservation than the rest of the proteins. The transmembrane regions show high percentages of identical amino acids, except for helices three and six. The transmembrane helices seven to nine and the connecting extracellular loop 4 and intracellular loop 4 build a highly conserved area across TMEM16 from different families and different species.

**Fig 2:**
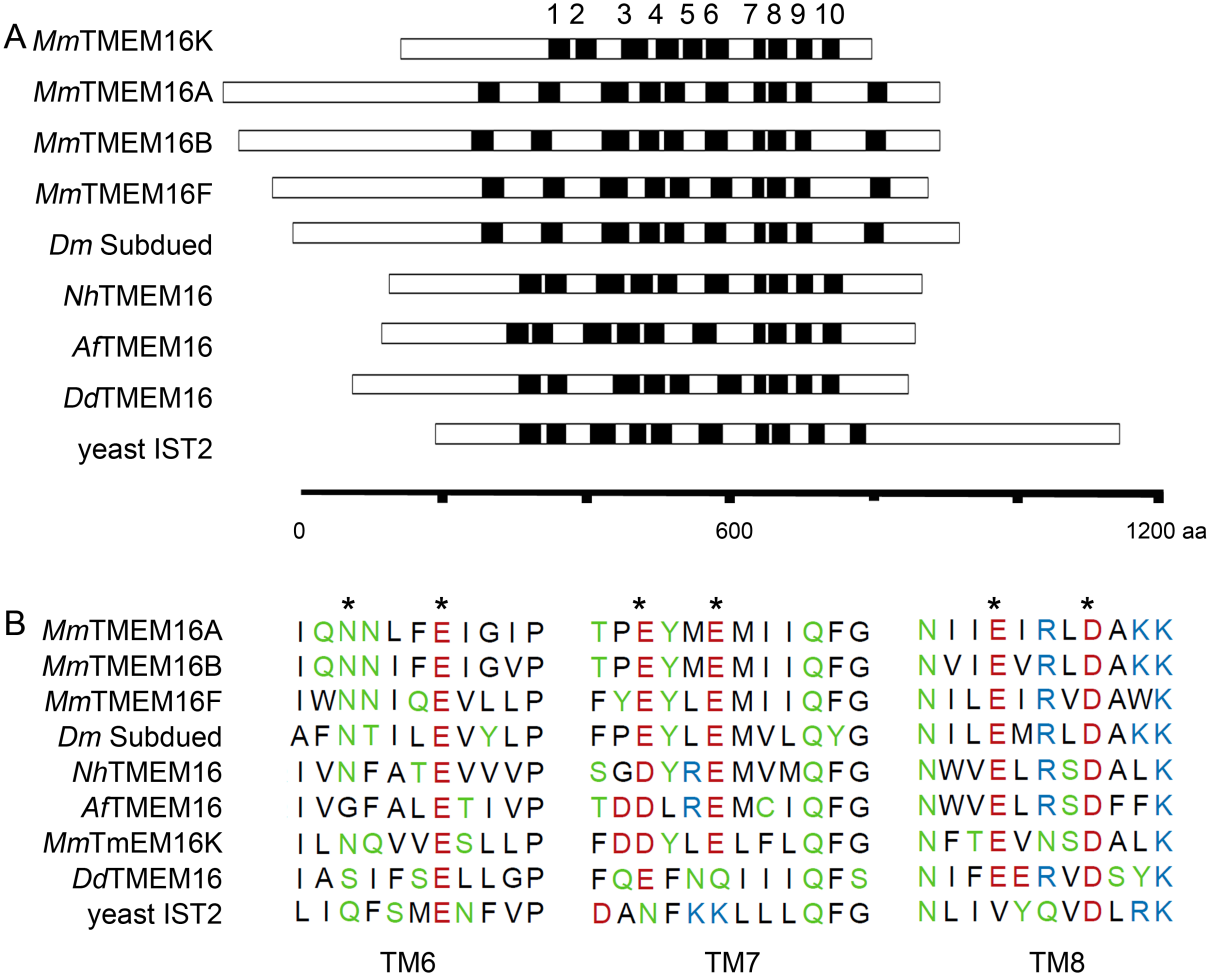
Protein domains in evolutionary early TMEM16 family members. (A) Schematic representation of topologies of selected TMEM16 proteins. Transmembrane domains (black) are numbered on top of the figure, loop regions and termini are represented by white bars. (B) Comparison of the Ca^2+^ binding motif identified in the crystal structure of *Nectria haematococca* TMEM16 (marked by *) with TMEM16 homologs from different species. Color scheme indicates amino acid polarity.

We next analyzed the membrane topologies of the well characterized Cl^-^ channels TMEM16A and TMEM16B from mouse, and compared them to evolutionary distant TMEM16 sequences for which functional information (*Aspergillus fumigatus*, *Nectria haematococca*, *Saccharomyces cerevisiae*) is available. In addition, we included sequence data from *Dictyostelium discoideum*, a social amoeba which belongs to the protozoan phylum of amoebozoa, a sister group to the animals and fungi that branched after the divergence of plants [42]. TMEM16A and TMEM16B have very similar topologies regarding the length of the loop domains connecting the 10 transmembrane (TM) segments, with relatively large extracellular loops between TM1/TM2 and TM9/TM10 (Fig 2A). TMEM16F and *Drosophila* Subdued have similar topologies, while these extracellular loop domains are shorter in TMEM16K and non-vertebrate TMEM16 isoforms. The length of the intracellular loops is more similar in different eukaryotes. The arrangement and the length of TM domains and loops in the ancient TMEM16 homologs from *D*. *discoideum* and yeasts are most similar to vertebrate TMEM16Ks.

Comparison of the Ca^2+^ sensing motif (Fig 2B) revealed conservation between mouse TMEM16 and Subdued, which was recently identified as a Ca^2+^-activated Cl^-^ channel [28]. The TMEM16-like sequence from *Nectria haematococca* also showed complete conservation of the motif [34], while the sequence from *Aspergillus fumigatus* displays an amino acid exchange in TM6 although scramblase activity of the protein is regulated by Ca^2+^ [33]. The *Dictyostelium* TMEM16 homolog showed amino acid exchanges in two of the proposed Ca^2+^ binding motifs, in yeast Ist2p all three motifs are significantly altered (marked by * in Fig 2B).

### Recombinant expression of TMEM16 from *D*. *discoideum*

Anion conductance and scramblase activity are not general features of all TMEM16 homologs. TMEM16A and TMEM16B do not seem to scramble phospholipids, and other TMEM16 proteins function as Ca^2+^-dependent phospholipid scramblases and not as a chloride channels. We therefore aimed to functionally investigate another early family member. *Dictyostelium* provides a valuable tool for studying the function of unknown genes in a system that is experimentally tractable and intermediate in complexity between the yeasts and the higher multicellular eukaryotes [43]. Moreover, compared to TMEM16A and TMEM16B, *Dictyostelium discoideum* TMEM16 (*Dd*TMEM16) showed a degree of amino acid conservation similar to the *Nectria haematococca* and *Aspergillus fumigatus* TMEM isoforms (*Dd*TMEM16 15.2%, *Nh*TMEM16 14.3%, *Af* TMEM16 15.8%).

**Fig 3:**
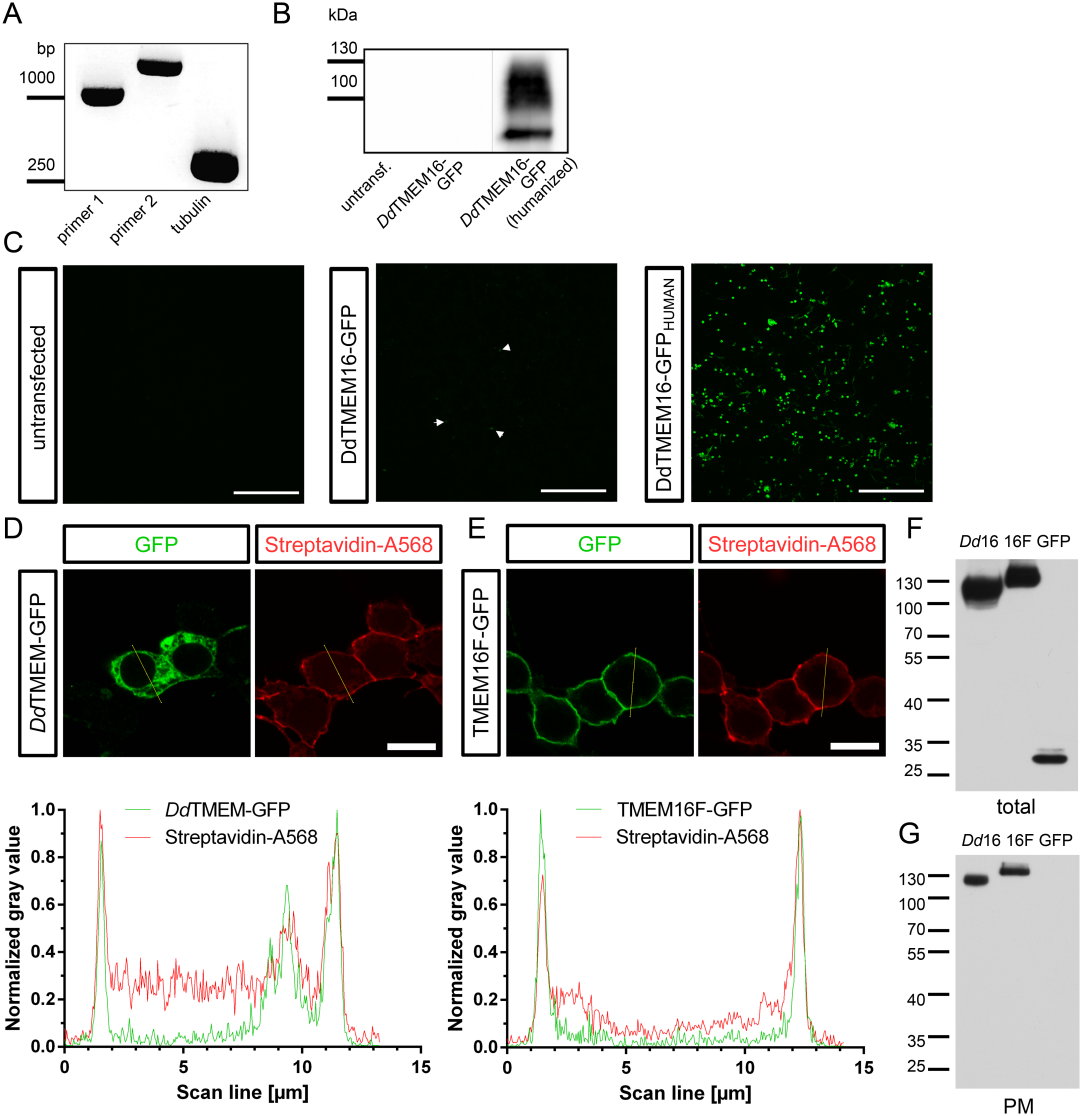
Recombinant expression of the TMEM16 homolog from *D*. *discoideum*. (A) RT-PCR with mRNA from axenically growing *Dictyostelium* cells showing expression of *Dd*TMEM16 with two different primer pairs. (B) Western blot of membrane preparations from HEK293 cells expressing *Dd*TMEM16 (*Dictyostelium* codon usage and human codon usage). (C) Expression of GFP-tagged *Dd*TMEM16 constructs in HEK293 cells. Shown are confocal pictures from the construct directly cloned from *Dictyostelium* mRNA and the construct coding for the same amino acid sequence using humanized codon-usage. Arrow heads mark green cells. Scale bars represent 300 μm. (D) Higher magnification of HEK293 cells expressing GFP-tagged *Dd*TMEM16 (green) after cell surface biotinylation, followed by labeling of biotin with streptavidin-coupled Alexa 568 (red). Diagram shows the normalized gray values in the respective channels along a scan line (yellow) through the cell, demonstrating localization of *Dd*TMEM16 at the cell surface. (E) HEK293 cells expressing GFP-tagged TMEM16F (green) and cell surface protein labeling as in (D), showing localization of TMEM16F at the cell surface. Scale bars represent 10 μm. (F) Western blot of whole cell lysates from HEK293 cells expressing *Dd*TMEM16-GFP (*Dd*16), TMEM16F-GFP (16F) and cytosolic GFP (GFP). (G) Western blot of cell surface proteins (after isolation of biotinylated proteins), showing presence of *Dd*TMEM16-GFP (*Dd*16) and TMEM16F-GFP (16F) at the plasma membrane (PM).

First, we tested if *Dd*TMEM16, which was so far only identified on a genomic basis by similarity, is actually expressed in *Dictyostelium* cells. We used mRNA from axenically growing *D*. *discoideum* and conducted RT-PCR analysis with two different primer pairs. Results showed that *Dd*TMEM16 is indeed expressed, as indicated by PCR products of the expected size (Fig 3A, primer pair 2 is intron-spanning).

We next asked whether this TMEM16 homolog also gives rise to a Ca^2+^-activated Cl^-^ conductance or to phospholipid scrambling. To address this question we cloned the full length *Dd*TMEM16 cDNA into a mammalian expression vector and tried to express the protein in HEK293T cells for electrophysiological analysis. Transfection of HEK293T cells did not result in the expression of the full length protein to a significant extent, few cells showed diffuse GFP fluorescence (Fig 3B and 3C). We hypothesized that the uncommon codon usage in *Dictyostelium* prevents the protein from successful translation in the mammalian expression system. The GC content of the *Dd*TMEM16 cDNA is extremely low (only 23%) compared to the average human gene with around 60% GC. We therefore synthesized the full length gene adapted to human codon usage for expression in mammalian cells. Transfection of HEK293 cells with the ‘humanized’ gene yielded expression of the *Dd*TMEM16-GFP construct (90 kDa + 27 kDa GFP), as indicated by the band between 100 and 130 kDa in Western blots (Fig 3B). To analyze the subcellular localization of *Dd*TMEM16-GFP, we labeled cell surface proteins of transfected HEK293 cells with membrane-impermeable Sulfo-NHS-SS-Biotin. For microscopical analysis biotin was visualized with fluorescently labeled streptavidin (Fig 3D) and compared to cells expressing TMEM16F-GFP, a well characterized mammalian TMEM16 family member (Fig 3E). Line scans through transfected cells showed localization of both proteins at the plasma membrane. In addition, transfected cells were lysed, and biotinylated proteins were isolated by adding streptavidin-coated beads (Fig 3G). Whole cell lysates were used to adjust for the amount of recombinantly expressed protein (Fig 3F). *Dd*TMEM16-GFP and TMEM16F-GFP were detected in the fraction of isolated plasma membrane proteins, while cytosolic GFP was not.

### Recombinant *D*. *discoideum* TMEM16 is not a chloride channel

**Fig 4:**
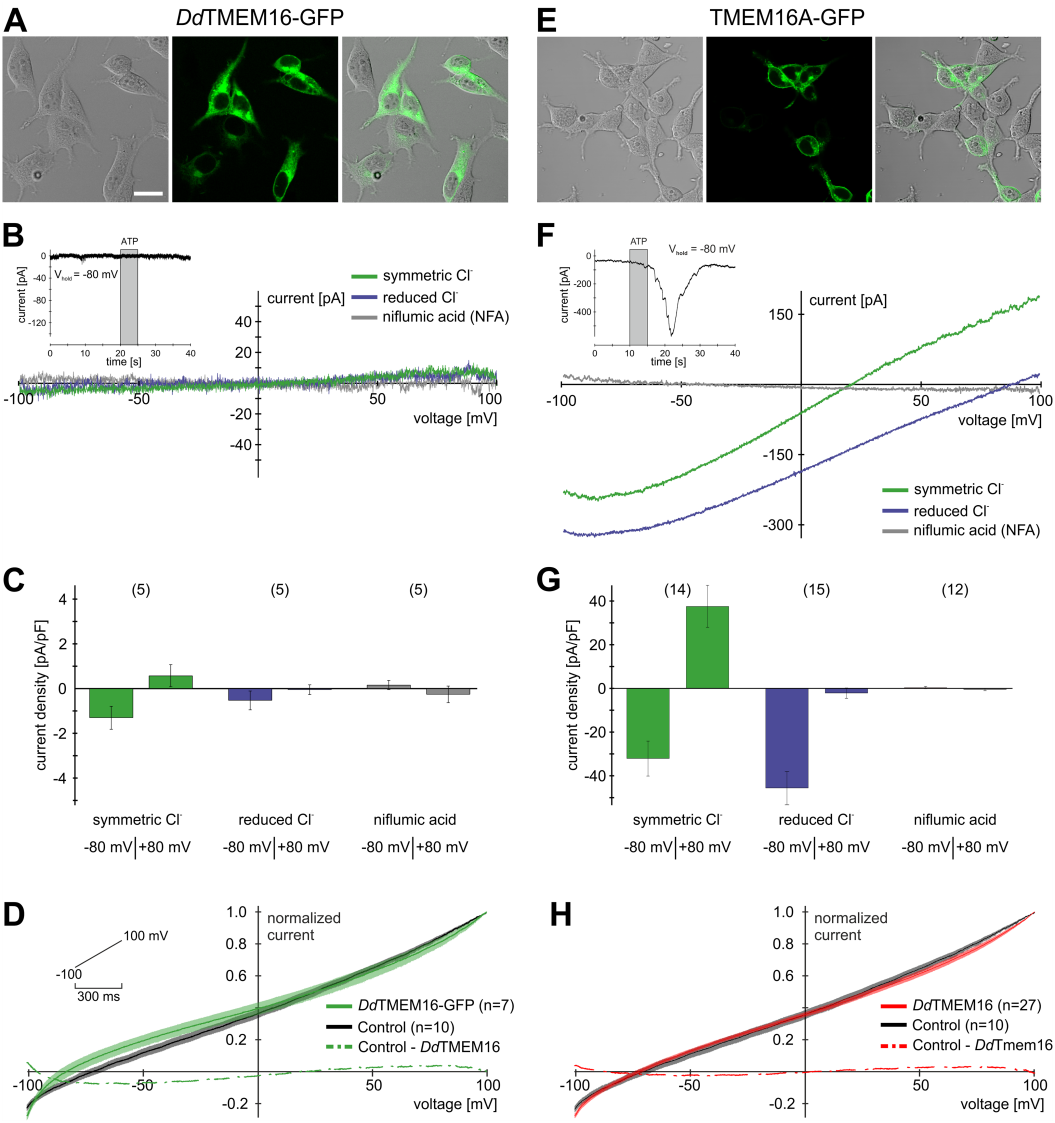
Recombinant *Dd*TMEM16 does not from a chloride channel. **(A)** *Dd*TMEM16-GFP is expressed in the plasma membrane of HEK293T cells. (**B)** In ramp protocols ranging from -100 mV to +100 mV, ATP stimulation fails to activate chloride currents. Accordingly, no shift in reversal potential upon reduction of extracellular chloride is observed and niflumic acid has no effect. Inset: ATP application (100 μM, 5 s, grey bar) during continuous recording (V_hold_ = -80 mV) does not induce current. By contrast, ratiometric Ca^2+^ imaging reveals substantial elevations in cytosolic Ca^2+^ upon ATP exposure (red curve / axis). [Ca^2+^] was calculated according to Grynkiewicz *et al*. [40]. (**C)** Maximal current densities at -80 mV and +80 mV (mean ± SEM). Bar chart quantification reveals no ATP (100 μM) dependent currents amplitudes under symmetric (n = 5) or reduced chloride (n = 5) conditions, or in presence of niflumic acid (n = 5). (**D**) Average normalized current-voltage curves recorded from both *Dd*TMEM16-GFP expressing (n = 7, green) and control (n = 10, black) cells after break-in. Inset depict the voltage ramp protocol. The dotted green trace results from digital subtraction. (**E)** TMEM16A-GFP is expressed in the plasma membrane of HEK293T cells. (**F)** Stimulation with 100 μM ATP activates chloride currents in patch-clamp experiments. In ramp protocols ranging from -100 mV to +100 mV the reversal potential is shifted upon reduction of extracellular chloride and currents are inhibited by niflumic acid. Inset: ATP application (5 s, grey bar) during continuous recordings (V_hold_ = -80 mV) results in a negative current. (**G)** Maximal current densities at -80 mV and +80 mV (mean ± SEM) in presence of symmetric chloride (n = 14), reduced chloride (n = 15) and niflumic acid (n = 12). The current densities of TMEM16A are significantly larger compared to DdTMEM16 (student’s t-test, p < 0.01). (**H**) Average normalized current-voltage curves recorded from both *Dd*TMEM16 (untagged) expressing (n = 27, red) and control (n = 10, black) cells after break-in. The dotted red trace results from digital subtraction. **S**cale bar represents 20 μm.

To investigate whether *Dd*TMEM16 functions as a Ca^2+^-activated ion channel, we analyzed the electrophysiological properties of *Dd*TMEM16 in HEK293T cells (Fig 4). Cells were transfected with humanized *Dd*TMEM16-GFP or mouse TMEM16A-GFP as a positive control (Fig 4A and 4E), and whole-cell patch-clamp recordings were performed from both fluorescently labeled and control cells. First, cells overexpressing either TMEM16 isoform were stimulated with ATP to trigger the release of Ca^2+^ from internal stores via activation of endogenous P2Y receptors [1]. In continuous recording mode (holding potential V_hold_ = -80 mV), we observed a robust inward current in TMEM16A-expressing cells, but not after transfection with *Dd*TMEM16 (Fig 4B and 4F, insets). To analyze corresponding Ca^2+^ elevations upon ATP stimulation, we performed ratiometric Ca^2+^-imaging experiments in HEK293T cells as described [40] and calculated maximum average concentrations of ~3 μM Ca^2+^ after ATP stimulation (100 μM; Fig 4B, inset). To test whether *Dd*TMEM16 is sensitive to higher intracellular Ca^2+^ concentrations we diffusion loaded the cells with 200 μM Ca^2+^ via the pipette solution and calculated average currents (mean ± SEM) from individually normalized current–voltage curves (Fig 4C) under symmetric chloride conditions (***S***_***2***_). We measured essentially identical currents in transfected and non-transfected cells (n = 12–19), both immediately after break-in and throughout the course of several minutes, indicating that the absence of currents upon ATP stimulation in *Dd*TMEM16 expressing cells (Fig 4B) does not result from low Ca^2+^ affinity. Moreover, the currents recorded from both transfected and control cells are essentially linear and reverse at approximately -75 mV.

Next, Voltage ramp recordings were performed from -100 mV to +100 mV at 500 ms duration using different extracellular solutions (***S***_***2***_, ***S***_***3***_, ***S***_***2***_+ niflumic acid, Fig 4B and 4F). For quantification, we calculated average current densities at -80 mV and +80 mV (Fig 4D and 4G). Under symmetric chloride conditions (***S***_***2***_), current densities for TMEM16A were -32.1 ± 8.0 pA/pF and 37.6 ± 9.6 pA/pF (n = 14) for 100 μM ATP. Indicating dose dependence, 10 μM ATP evoked -22.3 ± 6.6 pA/pF (-80 mV) and 26.5 ± 4.5 pA/pF (80 mV; n = 23), respectively (data not shown). By contrast, only minor currents were detected for *Dd*TMEM16 (-1.3 ± 0.5 pA/pF and 0.6 ± 0.5 pA/pF (n = 5). Under reduced chloride conditions (***S***_***3***_), current densities elicited by 100 μM ATP were -45.6 ± 7.7 pA/pF and -2.2 ± 2.4 pA/pF (n = 15) for TMEM16A, whereas 10 μM ATP evoked -43.9 ± 3.9 pA/pF (-80 mV) and 2.7 ± 4.0 pA/pF (80 mV; n = 15), respectively (data not shown). Again, negligible currents were recorded in cells transfected with *Dd*TMEM16 (-0.5 ± 0.4 pA/pF (-80 mV) and -0.1 ± 0.2 pA/pF (80 mV; n = 5)). Treatment with niflumic acid (300 μM), a known chloride channel blocker, strongly reduced TMEM16A currents at both potentials and stimulus ATP concentrations: 0.4 ± 0.5 pA/pF (-80 mV) and -0.5 ± 0.5 pA/pF (80 mV, 100 μM ATP; n = 12), 1.2 ± 0.4 pA/pF (-80 mV) and -0.9 ± 0.4 pA/pF (80 mV, 10 μM ATP; n = 21; data not shown) confirming that TMEM16A acts as a Ca^2+^-activated chloride channel. However, we did not observe significant inhibition of residual Ca^2+^-activated chloride currents in cells transfected with *Dd*TMEM16, indicating that *Dd*TMEM16 does not act as a Ca^2+^-dependent chloride channel.

To ensure that the lack of chloride conductance is not due to the addition of the GFP tag, we cloned a pIRES vector which promotes low level coexpression of ‘free’ cytosolic GFP for identification of transfected cells expressing untagged *Dd*TMEM16. To test whether untagged *Dd*TMEM16 is sensitive to high intracellular Ca^2+^ concentrations we again diffusion loaded the cells with 200 μM Ca^2+^ via the pipette solution and calculated average currents (mean ± SEM) from individually normalized current–voltage curves (Fig 4H) under symmetric chloride conditions (***S***_***2***_). Similar to the results obtained from expression of the tagged protein (Fig 4D), we measured essentially identical currents from cells expressing the untagged *Dd*TMEM16 (n = 27) and non-transfected cells (n = 10), both immediately after break-in and throughout the course of several minutes, indicating that the absence of currents in *Dd*TMEM16-GFP expressing cells (Fig 4B–4D) does not result from the presence of the GFP tag.

### Phospholipid scrambling by *Dictyostelium* TMEM16

We then addressed whether *Dd*TMEM16 acts as a phospholipid scramblase as do TMEM16F and TMEM16 homologs from fungi. We therefore investigated whether heterologous expression of *Dd*TMEM16 leads to Phosphatidyl-Serine (PtdSer) exposure on the outer leaflet of the plasma membrane. PtdSer exposure was measured by fluorescence imaging of Alexa Fluor 568-conjugated Annexin V, a well-established PtdSer probe which binds PtdSer with rapid kinetics [19, 44]. Imaging methods were chosen for the analysis of Annexin V binding, since damage of the cellular membranes of adherently growing HEK293 cells upon dissociation from the culture dish lead to PtdSer exposure irrespective of any prior treatment or transfection (data not shown).

**Fig 5:**
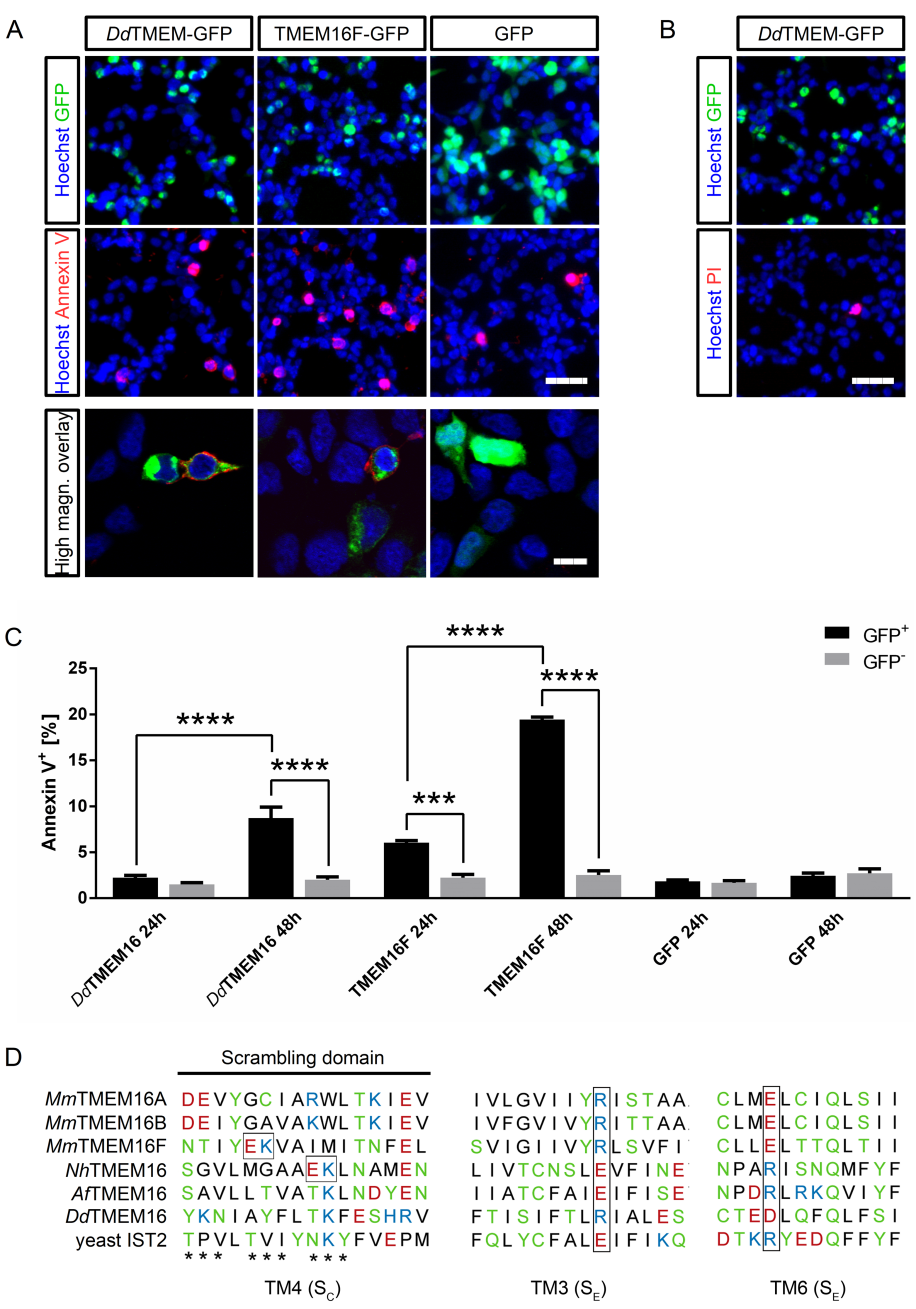
*Dd*TMEM16 functions as phospholipid scramblase. (A) HEK293 cells expressing *Dd*TMEM16-GFP, TMEM16F-GFP or GFP (green), co-stained with Annexin V-A568 (red) and Hoechst 33342 (blue) 48 h after transfection. Scalebar represents 50 μm. High magnification pictures, scale bar represents 10 μm. (B) HEK293 cells expressing *Dd*TMEM16-GFP (green), co-stained with propidium iodide (red) and Hoechst 33342 (blue) 48 h after transfection. Scale bar represents 50 μm. (C) Quantification of Annexin V-positive cells shown in (A) 24 h and 48 h after transfection. Data from three individual experiments (n = 3) are given as mean ± standard error of the mean (SEM). Black bars represent expressing (GFP-positive) cells, grey bars represent non-expressing (GFP-negative) cells from the same transfected cell culture dishes. P values were calculated using two-way ANOVA and Tukey’s multiple comparisons test. Asterisks represent multiplicity-adjusted P values (triple asterisk, P < 0.001; quadruple asterisk, P < 0.0001). (D) Scrambling domain according to [19] and [45]. The region associated with phospholipid scrambling in TMEM16F [19] is marked by asterisks. The cytosolic (S_C_) and extracellular sites (S_E_) of contact of the lipid headgroup with NhTMEM16 [45] are labeled, crucial amino acids are highlighted by brackets. *Dd*TMEM16 is aligned with sequences of mouse TMEM16A, TMEM16B and TMEM16F, *Nh*TMEM16, *Af*TMEM16, and yeast IST2. TM, transmembrane domain. Color scheme indicates amino acid polarity.

We analyzed PtdSer exposure in cells expressing *Dd*TMEM16-GFP, TMEM16F-GFP, and GFP only, and compared the expressing cells (GFP^+^) with non-expressing cells (GFP^-^) of the respective samples (Fig 5A and 5B). Cells expressing GFP only did not show a significant difference in PtsSer exposure compared to the non-expressing cells in the same dish (24 h, multiplicity-adjusted P value > 0.9999; 48 h, P > 0.9999). Also, a longer expression time (48 h) did not lead to PtsSer exposure in GFP-expressing cells (24 h vs. 48 h: expressing cells, P = 0.9977; non-expressing cells, P = 0.8820). Expression of *Dd*TMEM16-GFP did not have a significant effect after 24 h when compared to non-expressing cells in the same dish (P = 0.9895) and compared to GFP-expressing cells (P > 0.9999). 48 h after transfection, expression of *Dd*TMEM16-GFP lead to exposure of PtsSer (*versus* non-expressing cells in the same dish, P < 0.0001; *versus* GFP only expressing cells, P < 0.0001; *versus* 24 h *Dd*TMEM16-expression, P < 0.0001). TMEM16F-GFP expression induced a significant PtsSer exposure already after 24 h (*versus* non-expressing cells of the same dish, P = 0.0002; *versus* GFP only expressing cells, P < 0.0001). Similar as observed for *Dd*TMEM16, prolongation of the expression time increased the PtsSer exposure also in TMEM16F-GFP expressing cells (*versus* 24 h TMEM16F-expressing cells, P < 0.0001; *versus* non-expressing cells of the same dish, P < 0.0001; *versus* GFP only expressing cells, P < 0.0001).

Annexin V labeling in *Dd*TMEM16-expressing cells was not caused by apoptosis, since most cells were not labeled with propidium iodide, which can only penetrate damaged cell membranes (Fig 5B). The localization of the scrambling domain has been identified by mutation analysis in mouse TMEM16F [19] and by computational methods in *Nh*TMEM16 [45]. We therefore compared the domains shown to be important for lipid scrambling in TMEM16F and *Nh*TMEM16 with *Af*TMEM16, which also scrambles phospholipids, *Dd*TMEM16, and with TMEM16A, TMEM16B and IST2P, which do not scramble phospholipids (Fig 5D).

The alignment shows amino acid differences in the scrambling domain between *Dd*TMEM16 and TMEM16F and *Nh*TMEM16. The cytosolic contact site of the lipid headgroup with *Nh*TMEM16 (S_C_) overlaps with the scrambling domain in TMEM16F (Fig 5D). The charged glutamate and lysine of the S_C_ site are proposed to facilitate dipole stacking of the headgroup into the hydrophilic groove [45], and are present only in the scramblases, although at different positions in the helix. *Dd*TMEM16 and *Af*TMEM16 lack the glutamate and lysine pair, but have the glutamate replaced by threonine. Since *Af*TMEM16 is known to scramble phospholipids, one could speculate that the hydroxyl group of the threonine might be sufficient for headgroup coordination. The extracellular site of contact (S_E_) is composed of oppositely charged residues in transmembrane helices 3 and 6. The position of the basic and acidic residue is switched in fungal compared to mouse proteins; in this position *Dd*TMEM16 has the same arrangement of these amino acids as TMEM16F. This site is likely important for lipid permeation and ion conduction [45], and present in all homologues included in this alignment. Taken together, based on this sequence alignment *Dd*TMEM16 could also have a scrambling domain.

### Phospholipid scrambling by *Dd*TMEM16 depends on the intracellular Ca^2+^ concentration

**Fig 6:**
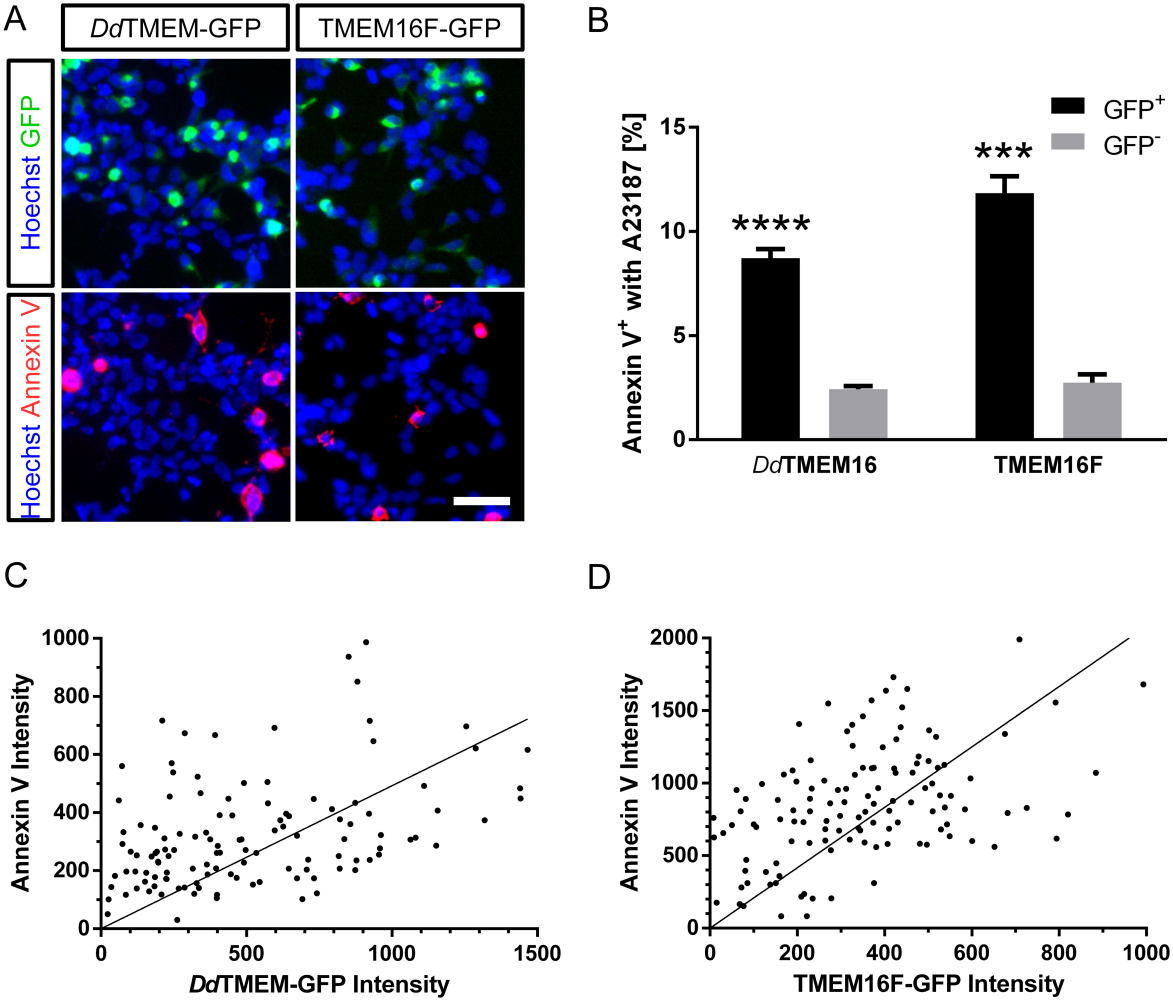
Scrambling by *Dd*TMEM16 depends on calcium. (A) A23187-stimulated HEK293 cells expressing *Dd*TMEM16-GFP or TMEM16F-GFP (green) one day after transfection, co-stained with Annexin V-A568 (red) and Hoechst 33342 (blue). Scalebar represents 50 μm. (B) Quantification of Annexin V-positive cells shown in (C). Data from n = 4 (*Dd*TMEM16) or n = 3 (TMEM16F) individual experiments are given as mean ± SEM. P values were calculated using unpaired t tests (triple asterisk, P < 0.001; quadruple asterisk, P < 0.0001). (C, D) Correlation between expression levels (mean grey values in the respective images) of *Dd*TMEM16 (C) and TMEM16F (D) with Annexin V-A568 binding. Data points in scatter plot represent single cells expressing *Dd*TMEM16-GFP (n = 122) or TMEM16F-GFP (n = 124). Straight lines are fitted to the data by linear regression, with Pearson’s correlation coefficients r = 0.40 (*Dd*TMEM16-GFP) and r = 0.44 (TMEM16F-GFP), respectively.

We next investigated whether intracellular Ca^2+^ elevation affects scramblase activity. PtdSer exposure in HEK293 cells expressing *Dd*TMEM16-GFP or TMEM16F-GFP was analyzed 24 h after transfection as longer expression already induced PtdSer exposure in a substantial percentage of cells. TMEM16F-GFP, which has already been shown to cause Ca^2+^-dependent PtdSer exposure [14, 46, 47], was used as a positive control. Intracellular calcium was elevated by incubation of the cells in 3 μM A23187, a divalent cation ionophore, followed by washout of A23187 and addition of 5 mM Ca^2+^ to initiate Ca^2+^-dependent scrambling [14, 19] (Fig 6A). Both *Dd*TMEM16-GFP and TMEM16F-GFP expressing cells showed an increase in PtdSer exposure after Ca^2+^ elevation compared to non-expressing cells in the same dishes (Fig 6B). The increase in PtdSer exposure was significantly higher in cells with high elevated Ca^2+^ concentration (incubated in A23187) compared to control cells with normal Ca^2+^ concentration (incubated in DMSO only). These data show that PtdSer exposure in *Dd*TMEM16-expressing cells was stimulated by Ca^2+^.

To assess the relationship between TMEM16 expression and PtdSer exposure, we quantified fluorescence intensities from *Dd*TMEM16-GFP and bound Annexin V-A568 in individual, A23187-stimulated cells (Fig 6C), and compared the results to cells expressing TMEM16F-GFP (Fig 6D). We found a moderate positive TMEM16/PtdSer correlation for both *Dd*TMEM16-GFP and TMEM16F-GFP that is shaped by the combinational variation between TMEM16 expression and PtdSer exposure, similar as described before for TMEM16F [19].

## Discussion

Members of the TMEM16 family are found throughout the eukaryotic kingdom. Mammals have 10 gene family members, whereas other animals have distinctly fewer. The cephalochordate amphioxus has six genes, insects have five to six genes, and *C*. *elegans* has only two genes. An exception is the presence of five genes in Cnidaria, which seems to be part of a wider trend for relatively large ion channel families in these species, such as voltage-gated potassium channels [48, 49], Nav_2_ channels [50] and Cav channels [51]. Most other eukaryotic species, such as fungi, plants, algae, and single-celled eukaryotes have single TMEM16 genes.

Mammalian TMEM16A and TMEM16B are Ca^2+^-activated Cl^-^ channels with important physiological functions. Likewise, Subdued, a *Drosophila* TMEM16 which is ~35% identical to mammalian TMEM16A and TMEM16B, displays characteristics of classic Ca^2+^-activated Cl^-^ channels [28], showing that some biophysical properties are evolutionarily conserved. TMEM16F and other mammalian TMEM16 family members are important for Ca^2+^-dependent phospholipid scrambling [14], the bidirectional transport of phospholipids between membrane leaflets.

One approach to address the question whether TMEM16 protein functions are evolutionary conserved is the analysis of TMEM16 homologs from simple eukaryotic species. Recent findings on an ancestral TMEM16 homolog from *Aspergillus fumigatus* support the hypothesis that the dual function of TMEM16 proteins might be conserved. Reconstituted *Af*TMEM16 builds non-selective channels of large conductance in planar lipid bilayers, which show low activation probability and only weak Ca^2+^ dependence. The protein, however, scrambles phospholipids [33]. On the other hand, the TMEM16 homolog Ist2p from yeast does not seem to fulfill any such functions. It plays a role in salt balance [52], and the cellular functions that are dependent on Ist2p are membrane trafficking and mRNA localization. Cells expressing yeast Ist2p together with P2Y receptors demonstrated whole-cell chloride currents, leading to the hypothesis that Ist2p enables Ca^2+^-dependent activation of a Cl^-^ channel already present in HEK293 cells [53].

Analysis of the TMEM16 homolog from *Nectria haematococca* showed that the protein functions as a phospholipid scramblase, but does not seem to have ion conductance properties when expressed in HEK293 cells or when reconstituted in lipid bilayers [34]. In a following report [35], *Nh*TMEM16 was found to mediate both ion and lipid transport with properties closely resembling those of *Af*TMEM16. The differences between both reports were attributed to the specific lipid compositions used in the reconstitution experiments and to the constructs used in the cell-based assays [35], showing that the function of TMEM16 proteins is extremely susceptible to the experimental conditions. Using whole-cell patch-clamp we demonstrate here that recombinantly expressed *Dd*TMEM16 does not form a functional Ca^2+^-activated ion channel. It is unlikely that poor plasma membrane expression of *Dd*TMEM16-GFP causes this lack of Cl^-^ conductance since plasma membrane localization was verified by confocal microscopy. Moreover, vertebrate TMEM16D-K are all able to produce transient Ca^2+^-activated Cl^-^ currents when expressed in HEK293 cells, although TMEM16H-K were mostly retained in the cytosol [54]. The presence of the GFP tag was not responsible for the lack of chloride conductance in HEK293 cells, since expression of the non-tagged *Dd*TMEM16 has not led to ion transport across the membrane either. However, it is possible that ion channel function, similar as in the case of *Nectria haematococca*, depends on a proper *Dictyostelium*-specific lipid environment. Moreover, we cannot exclude that *Dd*TMEM16 is activated by intra- or extracellular ligands other than Ca^2+^.

Despite the lack of observable ionic currents, *Dd*TMEM16 was functional since it acted as a phospholipid scramblase. Expression in HEK293 cells resulted in increased plasma membrane labeling with fluorescently tagged Annexin V, similar to the labeling upon expression of TMEM16F. The finding that *Nh*TMEM16, *Af*TMEM16 and the mammalian TMEM16F simultaneously mediate phospholipid scrambling and ion transport lead to the hypothesis that lipid scrambling could always be associated with simultaneous non-specific ion transport in TMEM16 proteins. Since G protein-coupled receptors that mediate lipid flipping do not show nonselective ion transport [55], the combination of both transport functions was proposed to be specific for TMEM16 proteins [35]. The lack of ionic currents in our experiments does not disprove this hypothesis, but our results suggest the closer analysis of *Dd*TMEM16 to understand the function of TMEM16-type scramblases and ion channels.

The proteins from non-animal model organisms that were used to study TMEM16 functions are relatively close related in evolutionary terms, all three species are members of the ascomycota, a phylum of the kingdom of fungi. Moreover, all TMEM16 homologs studied so far belong to the opisthokont branch of the phylogenetic tree, which includes the animal and fungal kingdoms. An organism outside this group is *Dictyostelium discoideum*, a representative of the amoebozoa group that diverged from the metazoa at some point after the plants, but before fungi [42, 56]. This organism has a unique lifecycle with motile unicellular and multicellular stages offering a variety of phenotypes associated with different signaling pathways. The *Dictyostelium discoideum* genome contains many genes that are homologous to those in higher eukaryotes and are missing in *Saccharomyces cerevisiae*, and the TMEM16 homolog of *Dictyostelium* shows higher sequence similarities to mammalian TMEM16 proteins than the Saccharomyces Ist2p. *Dd*TMEM16 is therefore more basal in the eukaryotic tree than the previously studied homologs, but shows a similar or higher degree of conservation.

The life cycle of *D*. *discoideum* consists of a solitary growth phase followed by a social phase that is induced by starvation and begins with the cAMP-mediated aggregation of individual cells to form a multicellular slug [57]. As development progresses, the anterior tip of the slug rises to form a fruiting body, other slug cells differentiate into vacuolated stalk cells that finally support a spore head. Extracellular exposure of PtdSer occurs during stalk cell differentiation in *D*. *discoideum*, and there is a gradual increase in the number of cells with exposed PtdSer restricted to prestalk cells [58]. Since only presumptive stalk cells show this change in membrane asymmetry, PtdSer exposure seems to be an important regulator of early development in *D*. *discoideum*. Whether *Dd*TMEM16 is involved in stalk development remains to be investigated, and would require that the protein is present at the plasma membrane.

Our results indicate that TMEM16 proteins in evolutionary early organisms function as phospholipid scramblases. Cl^-^ conductance of TMEM16 homologs from different animals, vertebrates and invertebrates, therefore likely represents one way in which proteins have gained novel functions to meet the needs of complex organisms.

## Supporting information

S1 Fig Sequence logo plot of all TMEM16 sequences. (PDF)

S1 Sequences Amino acid sequences of the included TMEM16 homologes. (TXT)
