# Supplementary figures and images for: An ancestral TMEM16 homolog from Dictyostelium discoideum forms a scramblase
Source: PLoS One. 2018 Feb 14;13(2):e0191219. doi: 10.1371/journal.pone.0191219 (PMC5812556; doi:10.1371/journal.pone.0191219)

S1 Fig: Sequence logo plot of all TMEM16 sequences

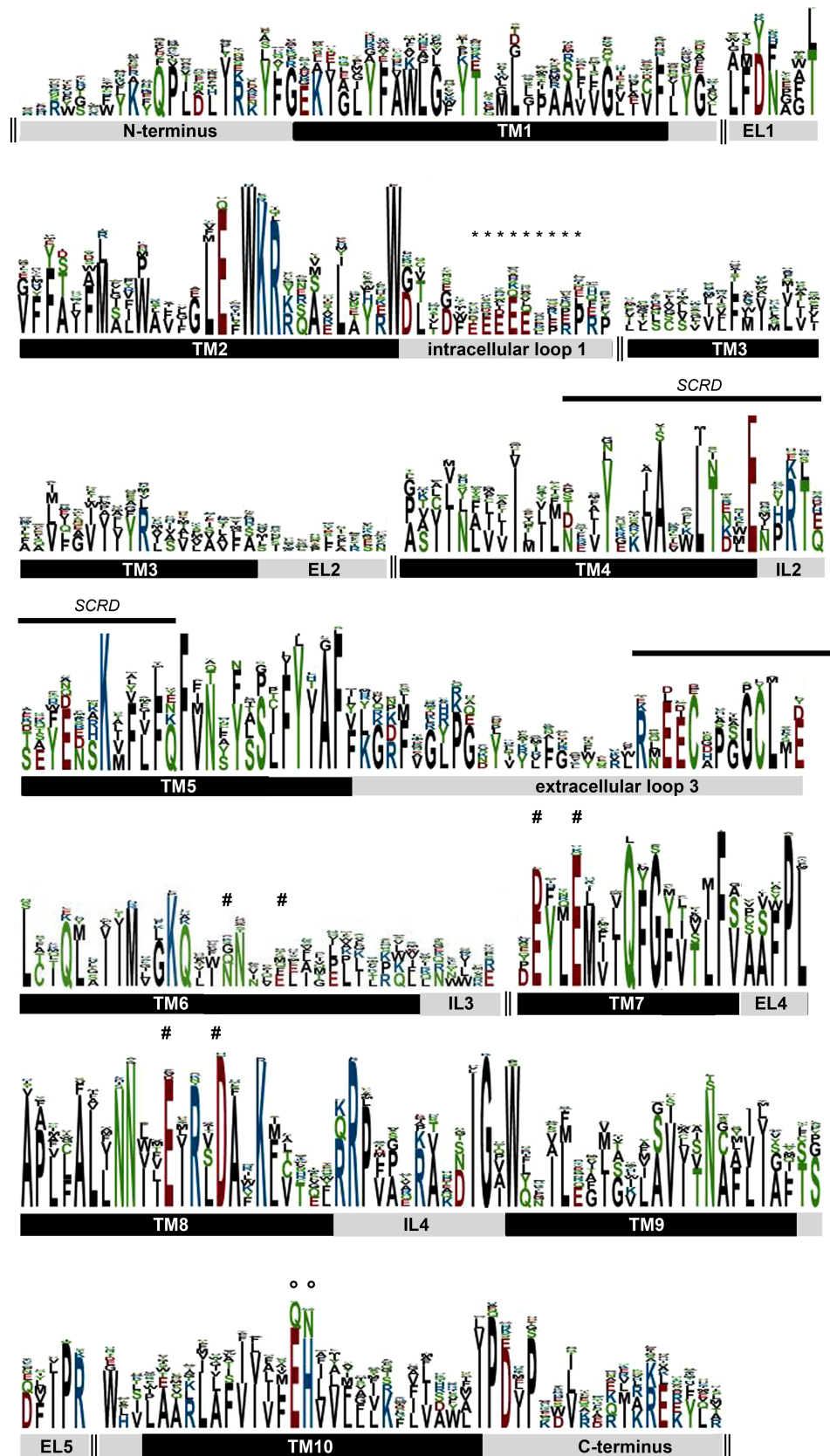

Supplement: S1 Fig — (PDF) [file pone.0191219.s001.pdf]
